# Effect of Combined Static Magnetic Field and Static Electric Field on the Supercooling Point and Quality of Beef

**DOI:** 10.3390/foods14183161

**Published:** 2025-09-10

**Authors:** Yujing He, Yuan Ma, Jingni Liu, Cenke Xiao, Lisha Liu, Yinying Li, Jiaxin Chen, Zhiying Quan

**Affiliations:** College of Food and Bioengineering, Xihua University, Chengdu 611743, China; 13458037231@163.com (Y.H.); 17844635105@163.com (J.L.); xiaocenke888@163.com (C.X.); lls2021523@163.com (L.L.); m15775436346@163.com (Y.L.); chenjx@mail.xhu.edu.cn (J.C.); 18784602607@163.com (Z.Q.)

**Keywords:** static magnetic field (SMF), static electric field (SEF), supercooling, beef

## Abstract

This study introduced a new low-temperature storage method that applies an additional lower strength static electric field (SEF) under the condition of a static magnetic field (SMF) to investigate the impact of magneto–electric coupling on the supercooling degree and quality of beef. The results showed that 7 mT-1 kV performs the best (−5.8 °C); the ability of SMF to maintain supercooling is less affected by SEF. Moreover, on the 15th day, magneto–electric coupling (7 mT-1 kV) outperformed SMF (7 mT) alone by reducing beef pH by 0.27, decreasing total viable counts (TVC) by 0.87 log CFU/g, maintaining TVB-N at only 12.5 mg/100 g, and limiting oxidative change, calpain activity, and shear force variation. Magnetic resonance imaging revealed that magneto–electric coupling treatment stabilized the T_2_ relaxation time in meat samples, effectively inhibiting immobilized water migration and promoting more uniform moisture distribution, highlighting its application potential as a low temperature preservation method.

## 1. Introduction

Beef is commonly preserved at low temperatures, between 4 °C and −80 °C, in states that range from unfrozen to frozen. Lower storage temperatures successfully prevent various biochemical reactions and microbial spoilage. Still, they can also cause the freezing of water in the meat, leading to the formation of irregularly sized ice crystals inside and outside the cells. This process can severely damage cell structures, including cell walls and membranes, resulting in deformation and rupture. Consequently, beef may experience substantial quality loss after thawing, such as impaired texture, reduced hydration retention, and diminished sensory attributes [1]. In order to mitigate ice crystal-induced damage to beef cell structures during low-temperature storage, current research primarily focuses on three strategic approaches: first, the use of supercooling storage technology to inhibit ice crystal formation [2]; second, the application of an SMF to lower the supercooling point and extend the duration of the supercooled state [3]; and third, the use of an SEF to reduce ice crystal size [4]. The combined application of SMF and SEF assisted superchilling technology shows promise for meat preservation, potentially enabling more precise control of the water molecule arrangement and cluster organization, enhancing the cell membrane stability, and further improving freezing efficiency.

Supercooling storage, a state where the temperature is maintained between the freezing point and the initial nucleation point (the lowest temperature reached prior to ice crystal formation), is the primary method for preventing ice crystal formation at its source. This temperature range prevents beef from freezing, significantly reducing microbial activity and extending shelf life. It also preserves the natural flavor and enhances the taste and quality of the stored products. However, supercooled water molecules are metastable and susceptible to freezing due to a variety of external factors. Maintaining a supercooled state is therefore critical to overcome the technical challenges associated with this method. The application of SMF has shown promise in stabilizing the supercooled state and minimizing the risk of food freezing caused by temperature fluctuations during supercooling storage [3]. Furthermore, SMF-assisted storage has been found to preserve food quality by enhancing the water-holding capacity of beef during both freezing [5] and supercooling [6]. Tang et al. [7] studied the effect of SMF on the freezing characteristics of pork at −30 °C (with a freezing rate of 4 °C/min). Their results indicate that SMF can lower the initial nucleation temperature of pork, increase the degree of supercooling, and sustain the supercooled state at lower temperatures. Magnetic field-assisted thawing of beef can improve efficiency and quality by shortening the thawing time, reducing drip loss, inhibiting lipid oxidation, and maintaining color and microstructure stability [8,9].

SEF primarily influences the nucleation temperature of the water molecules, inducing ice nucleation by raising the nucleation temperature [10]. By causing ice nucleation, reducing ice crystal size, and shortening the phase transition time, the electrostatic field minimizes structural damage to muscle microstructure caused by freezing. This results in increasing beef’s ability to hold water [11], and improving the quality of the frozen products [12,13]. The effect of SEF on the nucleation temperature is a trend of first increasing and then decreasing with higher electric field intensities [10,14,15]. Therefore, researchers usually select higher electric field intensities to promote ice nucleation effectively. SEF is also a promising non-heat technique that not only reduces the size of the ice crystals but also extends the shelf life of the goods by changing the distribution of bacteria within the product and reducing the number of spoilage bacteria populations [16,17].

Most experiments have found that the electric field has a better effect than the magnetic field in food preservation applications. Jiang et al. [18] investigated the effect of a high-voltage electric field on the quality of frozen broccoli and cauliflower, comparing it with a static magnetic field. Their findings showed the overall effect of a high-voltage electric (3 kV/cm) field on broccoli and cauliflower quality compared to the static magnetic field. Similarly, Hu et al. [19] also studied and compared the storage effects of electric and magnetic fields on pig spine, finding that SEF outperformed SMF in maintaining product quality. The roles of SEF and SMF in maintaining beef storage quality are fundamentally different. Magnetic fields can regulate ion transport and biofilm permeability in biological systems, thereby influencing cellular metabolism and enzyme activity. Electric fields can induce structural and functional changes in cells through mechanisms such as electroporation and electrophoresis. Given the distinct roles of magnetic and electric fields in preserving beef quality, this study investigated their synergistic effects during superchilled storage. Specifically, we analyzed the effects of an SEF and a magneto–electric coupling on the supercooling point of beef and compared their influences on quality attributes, tenderness, and water status.

## 2. Materials and Methods

### 2.1. Materials and Experimental Design

The beef (Guizhou yellow cattle,) comes from a stabilized slaughterhouse that processes a single breed of cattle of limited age and weight. Five Beef Longissimus Lumborum (BLL) muscle meat were purchased from Freshippo supermarket in Chengdu. All BLL (from five cattle) were immediately transferred to a portable cooler (6–8 °C) and then transported to the laboratory within 3 h after slaughtering. After removing fat, ligaments, and tendons, the beef samples were cut into 410 pieces measuring 2 × 2 × 2 cm^3^ (12 g, weighed on an electronic balance, AF224, Shanghai Hengping balance Scientific Instrument Co., Ltd., Shanghai, China).). The samples were wrapped in polyethylene film (PE) and placed in a 4 °C freezer (IINDUC Scientific Co., Ltd., Shuzhou, China) precooling for 8 h.

#### 2.1.1. Determination of Supercooling Temperature of Beef by SEF

**Figure 1:**
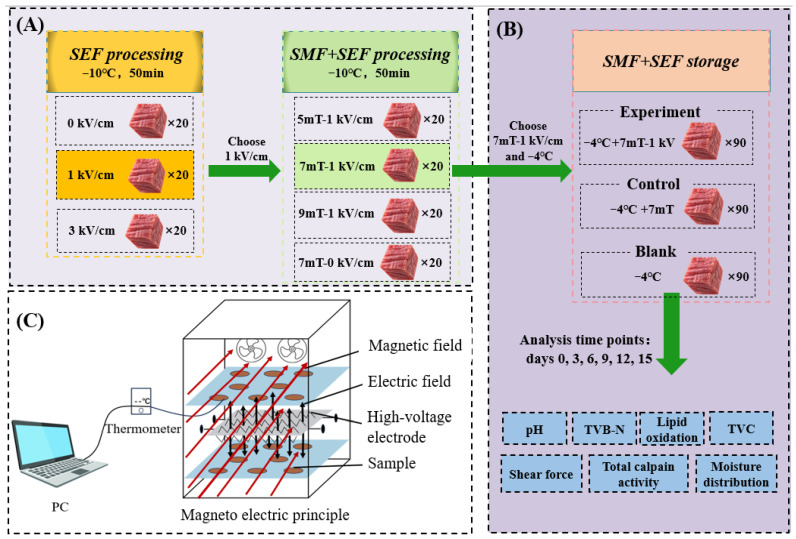
(**A**). The influence of electric and magnetic fields on the supercooling point of beef (**B**). The effect of electric and magnetic field assisted supercooling storage on the basic quality of beef. (**C**). Schematic diagram of the magneto–electric coupling system.

As shown in Figure 1A, 60 pre-cooled beef samples were randomly selected and divided into 3 groups of 20 each. The probe of a portable digital thermometer (GSP-6, Jiangsu Jingchuang Electric Co., Ltd., Xuzhou, China) was inserted into the center of each beef sample, which was placed in an adjustable magneto–electric coupling freezer (MEF-10, INDUC Scientific Co., Ltd., Wuxi, China) at −10 °C, and an SEF of 0 kV/cm, 1 kV/cm, and 3 kV/cm was applied to 3 groups of beef samples, respectively. The temperature change was recorded every 10 s for 50 min to ensure accurate temperature monitoring and to explore the effect of SEF on beef supercooling.

#### 2.1.2. Determination of Supercooling Temperature of Beef by SEF + SMF

As shown in Figure 1A, under the experimental results of Section 2.1.1, four groups of beef samples were randomly selected, with 20 samples in each group and placed in an adjustable magneto–electric coupling freezer (MEF-10, INDUC Scientific Co., Ltd., Wuxi, China) at −10 °C with SEF + SMF of 5 mT-1 kV/cm, 7 mT-1 kV/cm, 9 mT-1 kV/cm, and 7 mT-0 kV/cm applied. The probe of a portable digital thermometer was inserted into the center of each beef sample, and temperature changes recorded every 10 s for 50 min to ensure the accuracy of temperature monitoring and to explore the effect of SEF + SMF on beef supercooling. The core temperature of the BLL was monitored over time, with the temperature plotted on the *Y*-axis and time on the *X*-axis. In addition, the 20 supercooling points obtained from each of the seven groups were plotted as box plots.

#### 2.1.3. SEF + SMF Assisted Supercooling Storage

As shown in Figure 1B, 270 pre-cooled meat samples were randomly selected and divided into three groups. The meat was stored under different conditions based on the experimental results in Section 2.1.1 and Section 2.1.2—experimental group: −4 °C + 7 mT-1 kV/cm, control group: −4 °C + 7 mT, blank group: −4 °C. Changes in the basic physicochemical qualities of the beef were measured at 0, 3, 6, 9, 12, and 15 days. All samples were equilibrated at 4 °C for 6 h prior to indicator testing.

### 2.2. Determination of the pH

The experimental procedure was performed according to Wang et al. [20] A sample of 3 g was sliced into small pieces and placed in a plastic centrifuge tube. Then 30 mL of potassium chloride solution (0.1 mol/L) was added. The mixture was chopped and stirred using an adjustable high-speed homogenizer (FSH-2A, 7000 r/min, 30 s) until it reached a uniform broth-like consistency. The pH of the filtrate was measured at 25 °C using a previously calibrated pH meter (PHSJ-3F, INESA Scientific Instrument Co., Ltd., Shanghai, China). Each experiment was repeated 3 times. The pH meter and probe were calibrated at 25 °C with pH 4.0 and pH 6.8 standards.

### 2.3. Total Volatile Basic Nitrogen (TVB-N) Analysis

TVB-N content was determined using the semi-micro Kjeldahl method. The beef (5 g) was weighed and placed into a plastic centrifuge tube. After adding 37.5 mL of distilled water to this, the mixture was mixed thoroughly and allowed to stand for 30 min before filtering the mixture into a digestion tube. When using the automatic Kjeldahl nitrogen analyzer (K9840, Hanon Technologies Co., Ltd., Jinan, China), the following parameters were set: 0 mL of alkali and water, 30 mL of boric acid receiving solution, and a distillation period of 180 s. To perform distillation, 0.5 g of magnesium powder was added to the sample digestion tube and mixed thoroughly. Following sample collection, the distillate was titrated with 0.01 mol/L hydrochloric acid until the endpoint was reached (indicated by a color change in the solution from blue to purple red). Each experiment was repeated 3 times.

The volume of hydrochloric acid consumed was recorded for TVB-N calculation:

(1) $$X=\frac{V_{1}-V_{2}}{m}\times C\times 14\times 100$$

where *X*: TVB-N represents the value (mg/100 g) and below are the other symbols.

- *V*_1_: volume of hydrochloric acid consumed during titration (mL)
- *V*_2_: volume of hydrochloric acid consumed by the blank group during titration (mL)
- *C*: concentration of standard hydrochloric acid solution used in the titration process (mol/L)
- 14: the mass of nitrogen equivalent to 1.00 mL of hydrochloric acid standard solution (mg)
- *m*: mass of the sample.

### 2.4. Total Viable Counts (TVC)

The method for determining the TVC is based on the method description [21] as follows: 45 mL saline conical flasks containing 5 g of chopped beef samples were shaken using a shaker. In a sterile petri dish, 1 mL of the homogenized sample solution at various dilutions was added to the plate count agar medium and thoroughly mixed. The plate was inverted and incubated for 48 h at 36 ± 1 °C after solidification. Each experiment was repeated 3 times. Use the following formula to calculate *TVC*:

(2) $$N=\frac{C}{(n_{1}{+0.1n}_{2})d}$$

where *N* represents the number of colonies in the sample and below are the other symbols.

- *C*: sum of the number of colonies in the plates (plates containing the appropriate range of colony numbers)
- *n*_1_: number of plates at the first dilution (low dilution)
- *n*_2_: number of plates at the second dilution (high dilution)
- *d*: dilution factor (first dilution).

### 2.5. Lipid Oxidation Measurement

The method of lipid oxidation refers to Sobral, et al. [22]. Minced beef (5 g) was mixed thoroughly with 25 mL of 7.5% trichloroacetic acid (TCA) solution containing 0.1% EDTA-2Na. The mixture was homogenized using a homogenizer set to 6000 r/min for 1 min. After homogenization, the sample was centrifuged at 4 °C and 4000 r/min for 15 min. Then, 2 mL of the supernatant was aspirated and 2 mL of a 0.02 mol/L TBA solution added. The resulting solution was thoroughly mixed and heated in a boiling water bath for 30 min. Once the sample solution had cooled, 200 μL of the sample solution was taken and its absorbance measured at 532 and 600 nm using an enzyme-labeled instrument. Each experiment was repeated 3 times. The TBARS value, which represents the mass of malondialdehyde per kilogram of lipid oxidation solution, was calculated using the formula below:

(3) $$TBARS\left(\frac{mg}{kg}\right)=\frac{\left(A_{532}-A_{600}\right)\times 72.6\times 100}{155\times 10}$$

### 2.6. Determination of Shear Force

The measurement of shear force was determined as described by Hou et al. [23] with modifications. Two samples of meat were randomly selected from each of the three groups on days 0, 3, 6, 9, 12, and 15. The meat was sliced into 8 strips measuring 2 × 1 × 1 cm^3^, each strip (3 g) was weighed by an electronic balance (AF224, Shanghai Hengping Scientific Instrument Co., Ltd., Shanghai, China), then vacuum packed and boiled at 80 °C for 10 min. After allowing the strips to cool to room temperature, the shear stress of the beef perpendicular to the muscle fibers was measured using a texture analyzer (TA-XT PLUS, Stable Micro Systems, Godalming, UK). The measurement conditions were as follows: HDP-BSW probe, measurement speed at 1.5 mm/s, pre- and post-measurement speeds of 5 mm/s, trigger force of 20 g, and a distance of 30 mm. Each experiment was repeated 8 times.

### 2.7. Total Calpain Activity

The total calpain activity in beef samples were assessed as follows using the Bovine Calpain ELISA Kit (YJ061681, Shanghai Yuanjie Biotechnology Center, Shanghai, China).

### 2.8. Determination of Moisture Distribution and State

Beef samples were cut into pieces measuring 2 × 2 × 1 cm^3^ and analyzed using a low-field NMR analyzer (MesoMR23-040V1, Shanghai, China) with a phase-cycled Carr–Purcell–Meiboom–Gill sequence (CPMG) to measure the transverse relaxation T_2_. Parametric conditions: P_1_ = 1.152 μs, P_2_ = 22 μs, TD = 160,000, TW = 4000 ms, NS = 4, NECH = 4000. Each experiment was repeated 6 times. For cross-sectional magnetic resonance imaging of the samples, the imaging parameters were as follows: TR = 400 ms, TE = 20 ms, and mean = 8 ms. The imaging spectra obtained were processed using pseudo-color software. Each experiment was conducted in triplicate.

### 2.9. Data Analysis

A generalized linear model was used to analyze the physicochemical data. In Section 2.1.1 and Section 2.1.2, the strengths of electric and magnetic fields, as well as the freezing temperature and time, were used as fixed effects, while beef was considered as a random effect. In Section 2.1.3, Section 2.2, Section 2.3, Section 2.4, Section 2.5, Section 2.6, Section 2.7, Section 2.8 and Section 2.9, processing and storage days were treated as fixed effects, while beef was considered as a random effect. The interaction between fixed factors during processing and storage was also assessed. Statistical analysis was conducted using SPSS 27.0 software. Sample comparisons were made using ANOVA (repeated measure) with the Tukey test applied for mean comparisons (*p* < 0.05). The experimental data are expressed as mean ± SE to represent the central tendency and variability of the dataset. Significant changes between treatments were denoted by different letters, indicating variations based on pairwise comparisons. For graphical representation, Origin 2021 was employed to generate images and visualizations, ensuring clarity and precision in data presentation.

## 3. Results

### 3.1. Effect of Supercooling Point

**Figure 2:**
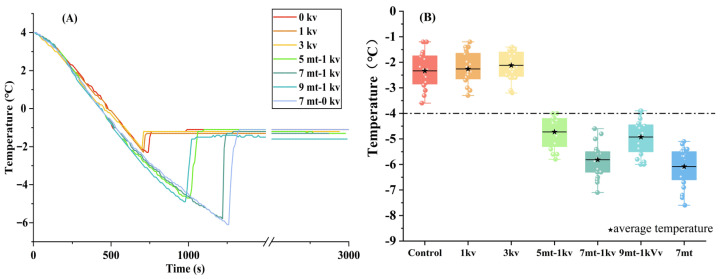
(**A**) Temperature–time curves of beef tenderloin under different freezing conditions. (**B**). Box point plots of supercooling points under different freezing conditions.

Figure 1C shows the technical principle of the magneto–electric coupling system, where the SMF is applied horizontally, and the SEF is generated by a pricked electrode and applied vertically. Figure 2A shows that SEF treatment alone has a certain effect on the nucleation temperature of beef, with a slight increase observed compared to the blank group. This effect can be attributed to the influence of the electric field on the free energy barrier during phase transition [24]. Specifically, the electrostatic field promotes the reorganization of water clusters by inducing directional alignment of the water molecular dipoles and strengthening hydrogen bonding along the field direction, thereby reducing the critical nucleation radius and decreasing the system’s Gibbs free energy to enhance the nucleation rate [25,26]. The observation aligns with the findings of Fallah-Joshaqani, et al. [27] who studied the influence of electrostatic fields (3.2 × 10^5^, 6.4 × 10^5^, 9.6 × 10^5^ V/m) on various freezing food parameters in physiological saline. Their results revealed that the nucleation temperature initially increased and subsequently decreased as the strength of the electrostatic field increased. This finding was in line with the experimental findings of [14]. In this study, the use of a weak static electric field exhibited a negligible effect on the nucleation temperature of beef. However, combining SMF or SEF + SMF lowered the supercooling point, enabling beef to remain unfrozen at lower temperatures. This effect is primarily attributed to the significant role of the static magnetic field in the freezing process. As water molecules are polar, the static magnetic field disrupts the framework of water clusters, induces rotation or vibration of the water molecules, lowers the supercooling point, increases the supercooling degree, and prevents ice nucleation in the beef [6,7,28].

Figure 2B illustrates the effects of magnetic and electric fields on the supercooling of beef. Box plots show the distribution of 20 supercooling points that were measured in each experiment. Under the conditions of 7 mT and 7 mT-1 kV, all data points fall below −4 °C. There is only a 0.3 °C difference between the average supercooling point at 7 mT (−6.1 °C) and 7 mT-1 kV (−5.8 °C). As a result, the temperature of −4 °C and the magnetic field electric field strength of 7 mT-1 kV were selected for subsequent experiments.

### 3.2. pH

**Figure 3:**
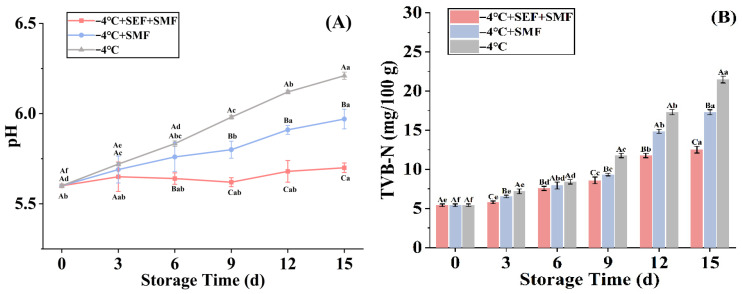

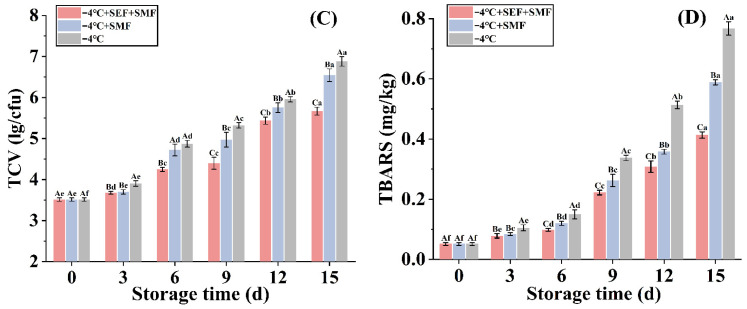
Changes in physicochemical indexes of beef under different storage conditions. (**A**) pH. (**B**) TVB-N. (**C**) TVC. (**D**) TBARS. Lowercase letters represent the significance of different days under the same treatment conditions, while uppercase letters represent the significance of different treatment groups under the same treatment conditions. Different letters indicate significant differences based on Tukey’s test (*p* < 0.05). Error bars depict the standard error of the mean.

The pH value significantly influences the color, tenderness, flavor, and shelf life of beef, making it a frequently used indicator for assessing the quality of meat products. Fresh beef typically has a pH range from 5.50 to 5.80 and gradually increases with storage medium—quality meat falls within the range of 5.80–6.09, and stale beef has a pH exceeding 6.10 [29,30]. On the first day of the experiment, the pH of the beef was measured as 5.60, within the acceptable range for fresh beef. It is evident from Figure 3A that as time went by, the pH values in the three treatment groups showed an upward trend, this is attributed to microorganisms breaking down the beef protein during storage, releasing alkaline nitrogen-containing chemicals such as ammonia and other biogenic amines that can cause a persistent rise in pH [31]. The −4 °C + SEF + SMF and −4 °C + SMF groups were 5.70 and 5.97 on the 15th day, respectively. In comparison to the beef on day 0, which satisfies the requirements for fresh beef, the pH value of the beef at −4 °C + SEF + SMF did not vary substantially. While low temperatures effectively inhibit metabolic enzyme activity and bacterial growth, thereby slowing pH rise, the combination of electric [32] and magnetic fields [33] with low temperatures was shown to enhance this effect. In this experiment, compared with low temperature and magnetic field, the combined effect of low temperature and electromagnetic field more effectively suppressed the pH value change of beef. By the fifteenth day, this combined approach successfully maintained the pH of the beef within the fresh range by simultaneously using electric and magnetic fields.

### 3.3. TVB-N

During storage, microorganisms and endogenous enzymes break down and use different nutrients in meat, leading to the accumulation of alkaline nitrogen-containing molecules such as ammonia and amines. This breakdown of proteins and other nitrogen-containing compounds significantly affects the product’s color and flavor and influences consumer purchasing decisions [34]. Total volatile alkaline nitrogen (TVB-N) serves as a common biomarker for the breakdown of proteins and amines, reflecting the extent of protein degradation and amine formation [34]. As TVB-N concentration increases, the freshness and storage quality of meat declines. According to the Chinese National Food Safety Standard (GB 2707-2016, 2016), fresh beef must not exceed 15 mg/100 g to ensure its freshness and quality [35].

As shown in Figure 3B, the TVB-N content increased across all treatment groups throughout the storage period. On the 12th day, the TVB-N content in the −4 °C + SEF + SMF group was 11.76 mg/100 g, while that in the −4 °C + SMF group was 14.84 mg/100 g, indicating that the freshness of the meat in the −4 °C + SMF group was lower than that in the −4 °C + SEF + SMF group. The results align with previous findings that storage under static magnetic fields (e.g., 4 mT and 10 mT) prolonged pork’s freshness, with TVB-N contents of 25.27, 13.46, and 15.47 mg/100 g observed for the control, MF-4, and MF-10 groups, respectively, after 8 days of storage in a static magnetic field (4 mT, 10 mT) [36]. Furthermore, Xu et al. [37] reported that continuous high-voltage electrostatic fields (CHVEF) effectively reduced TVB-N content in pork during freezing point storage, outperforming intermittent (IHVEF) and single-use (SHVEF) electrostatic fields. This reduction was attributed to the electrostatic field’s ability to inhibit various microbial and enzymatic activities. In this experiment, under magnetic field assisted ultra-low temperature storage at −4 °C, an additional 1 kV SEF could maintain the freshness of beef on the 15th day, and the TVB-N content in beef was less than 15 mg/100 g. These experimental results emphasize that the simultaneous use of SEF and SMF can improve the preservation of meat freshness compared to using them alone.

### 3.4. TVC Value

The total number of bacterial colonies is a crucial metric for evaluating beef quality as microorganisms significantly contribute to the role of meat deterioration. Figure 3C displays the live bacterial count in each treatment group over the storage period, showing an upward trend as storage duration increases. The condition −4 °C + SEF + SMF is more effective at controlling bacterial proliferation during storage than −4 °C + SMF alone; on the fifteenth day, the total number of colonies in beef treated with −4 °C + SEF + SMF was 5.67 lg (CFU/g), a reduction of 0.87 lg (CFU/g) compared to the SMF group. The three experimental conditions used in this experiment, low temperature, electric field, and magnetic field, affect the growth of microorganisms in three aspects. Low temperature significantly reduces the activity of microorganisms, delays their metabolic processes, and thus inhibits their growth, development, and reproduction [38]. External electric fields mainly disrupt cellular metabolism and physiological processes by promoting cytoplasmic leakage, microbial death, or changes in cell membrane permeability [16,39]. Similarly, SMF affects the permeability of cell membranes, which may lead to excessive permeability, resulting in microbial aging, nutrient leakage, and ultimately cell death [40]. In addition, SMF may affect DNA stability and oxidative free radical activity, leading to microbial developmental disruption [41]. Ko, Yang [42] reported that an SEF reduced microbial growth in chilled tilapia by tenfold compared to the control group. Tong et al. [43] discovered that an SMF (5 mT) significantly inhibited bacterial growth in refrigerated black sea bass. This is consistent with the results of this experiment. The SEF can disrupt microbial cell membrane structures through electroporation, leading to leakage of cellular contents and subsequent cell death [44]. Meanwhile, SMF can influence the electron spin state of free radical pairs, altering their recombination rate, and thereby affecting redox reactions and free radical-mediated cellular damage [45]. Together, they synergistically inhibit microorganisms.

### 3.5. Lipid Oxidation

Lipids are vital substances for human nutrition, serving as a primary energy source and providing crucial nutrients such as fat-soluble vitamins or important fatty acids. One of the key elements influencing the nutritional value of meat is lipid oxidation, producing compounds that adversely alter the meat’s color, texture, and odor, thereby reducing customer appeal [46]. To evaluate lipid oxidation, the thiobarbituric acid active substance (TBARS) assay is the most widely utilized of the many detection techniques [47].

As illustrated in Figure 3D, there was an increased trend in the malondialdehyde (MDA) levels across all treatment groups, indicating progressive lipid oxidation. You et al. [48] found that people who have received sensory training will detect an unpleasant taste when the TBARS value exceeds 0.5 mg/kg. On the 12th day, the TBARS values for the SMF and SEF + SMF dropped by 0.16 mg/kg and 0.20 mg/kg, respectively, compared to the −4 °C blank group (0.41 mg/kg). Notably, beef stored under electromagnetic field conditions for 15 days exhibited a TBARS value of 0.42 mg/kg, indicating that the beef’s quality remained acceptable and free from noticeable oxidative rancidity. However, under SMF conditions, beef already produced a noticeable unpleasant taste (0.58 mg/kg). Low temperatures are effective at inhibiting lipid oxidation [2], and this inhibitory effect was significantly enhanced (*p* < 0.05) with the application of SMF and SEF + SMF at low temperatures. Lipid oxidation was significantly inhibited in a large, yellow croaker stored at near-freezing temperatures with the application of high-voltage electrostatic fields (5, 8, and 16 kV/m). On the 21st day of storage, the MDA content of the control group and the HVEF groups (5 kV/m, 8 kV/m, and 16 kV/m) was 8.45, 3.55, 3.04, 2.48 mg/100 kg, respectively [49]. HVEF has been reported to significantly reduce lipid oxidation in tilapia during refrigeration, because the electrostatic induction phenomenon of HVEF makes the material surface charged, thereby reducing the frequency of contact between the fish and surrounding oxygen and slowing down the rate of lipid oxidation in fish meat [42]. However, contrasting findings were reported by Kantono et al. [50] where pulsed electric fields (PEF, 80–110 kV/m) applied to treat BLL increased the degree of fat oxidation. Faridnia et al. [51] suggested that PEF treatment, which promotes mechanical damage to beef muscle membranes caused by PEF, changes the cell structure promoting exposure to pro-oxidants and accelerating lipid oxidation.

### 3.6. Shear Force

**Figure 4:**
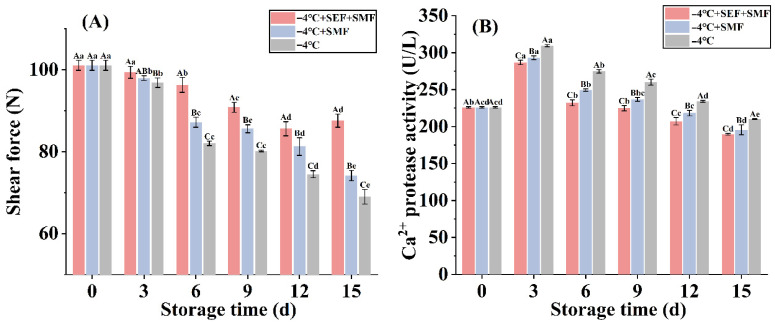
Changes in physicochemical indexes of beef under different storage conditions. (**A**). Shear force. (**B**) Ca^2+^ protease activity. Lowercase letters represent the significance of different days under the same treatment conditions, while uppercase letters represent the significance of different treatment groups under the same treatment conditions. Different letters indicate significant differences based on Tukey’s test (*p* < 0.05). Error bars depict the standard error of the mean.

The tenderness of meat overall amounts to the total amount of connective tissue and the structural condition of the myofibrillar proteins in muscles. Shear force is a measure of meat tenderness and serves as a reliable measure of tenderness. A higher shear force generally indicates tougher meat, while a lower shear force correlates with greater tenderness. Figure 4A illustrates the range in beef shear force over the storage period. The shear force of beef often exhibits a declining trend with storage. During storage, beef is exposed to microbial and endogenous enzymes, particularly calpain. These enzymes cause myofibrillar protein structure to be destroyed, leading to a decrease in shear force and an improvement in meat tenderness [52]. The −4 °C low-temperature group experienced the fastest loss in shear force, in part because of the action of endogenous proteases and microbes, as noted by Han et al. [53], and partly due to the formation of ice crystals at −4 °C. These ice crystals break down the myofibrillar structure of the meat, further contributing to the reduction in shear force after thawing [54]. The −4 °C + SEF + SMF group experienced the slowest decline in beef shear force, maintaining a shear force of 85.61 N on day 12, which was the same as the 4 °C + SMF group’s shear force on day 9. The findings align with the results of Lin et al. [3] who reported similar trends when storing beef in a static magnetic field, where the shear force of −4 °C + SMF was higher than that of the −4 °C blank group. In this experiment, applying an additional 1 kV electric field had a more significant effect on suppressing the shear force of beef. This may be because the SEF and SMF affect the activity of microorganisms and proteases in beef, so the calpain protease activity of beef was determined in the following experiments.

### 3.7. Ca^2+^ Protease Activity

The most popular enzyme for researching meat tenderness is the calpain system, making it the primary cause of meat tenderization [55]. Meat aging is dominated by μ-calpain and m-calpain, two distinct proteases that require varying Ca^2+^ activation concentrations. These enzymes are only active in their activated state [56] when exposed to the necessary calcium levels; the two calpain proteases gradually undergo autolysis, leading to a loss of enzymatic activity over time [57].

Figure 4B displays the total calpain activity measured in the beef samples, which first shows a rising trend before declining. On the third day, calpain was activated by the release of Ca^2+^, exhibiting a rising trend. Subsequently, the combined effects of SEF, SMF, low temperature, and autolysis caused its content to decrease. On the third day, the protease activity levels for the −4 °C + SEF + SMF, −4 °C + SMF, and −4 °C low-temperature groups were 286.69, 293.22, 309.47 units, respectively, the protease activity of 4 °C + SEF + SMF was 6.53 units lower than that of −4 °C + SMF; the superposition of the two fields’ effects may be the reason for SEF + SMF’s strongest calpain-inhibiting impact. As shown in Figure 1C, when using an SMF alone, only the vertical direction is effective. After applying SEF + SMF, both horizontal and vertical directions act simultaneously, which may also affect the activity of calpain in beef and the overall quality of beef throughout the storage period; this is also the reason why the joint use of SEF and SMF is more significant in maintaining beef quality The increase in total calpain activity leads to the destruction of the myofibrillar protein structure, resulting in a decrease in the shear force value of beef, which confirms the results of the previous section. In the study of Valdez-Miranda et al. [58], treatment with an SEF (6 kV/cm) significantly reduced the enzyme activity of polygalacturonase and pectin methylesterase in banana peels compared to the control group. However, Bhat, Morton [59] observed an increase in Ca^2+^ activity when applying PEF (5 kV, 90 Hz, 0.38 kV/cm and 10 kV, 20 Hz, 0.61 kV/cm) to beef. Yan et al. [60] found that HVEF (3 kV/cm) can inhibit the polyphenol oxidase activity of *Agaricus bisporus* and maintain high superoxide dismutase and catalase activity during storage. This suggests that the effects of EF on enzyme activity are highly dependent on the specific field parameters as well as the enzyme species. The SEF may modulate intracellular calcium ion concentrations—a key activator of calpain—and thereby regulate calpain activity through alterations in calcium homeostasis [61].

### 3.8. Water Distribution

**Figure 5:**
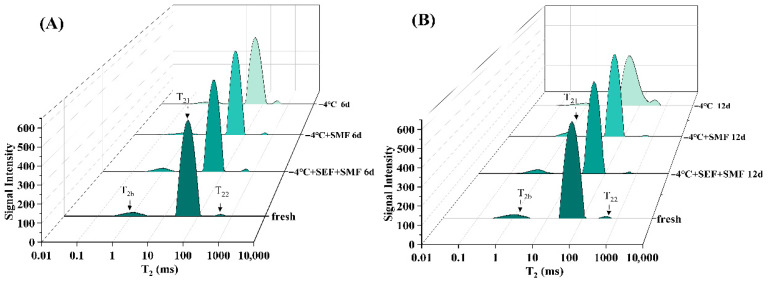

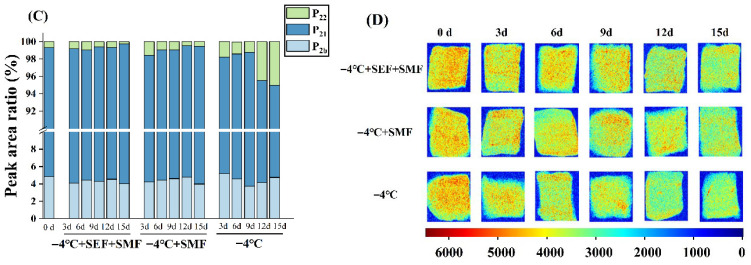
Effect of different storage conditions on the response signal of the T_2_ transverse relaxation peak in beef (**A**). Relaxation peak response signal on day 6. (**B**). Relaxation peak response signal on day 12. (**C**). Effect of different treatment groups on the percentage of peak area of lateral relaxation P2 of beef T2. (**D**). Effect of different treatment groups on H-proton imaging spectra of beef.

Low-field nuclear magnetic resonance (LF-NMR) is a rapid non-destructive technique for detecting moisture. The attenuation of nuclear magnetic resonance signals may typically be fitted into a distribution index comprising two to three separate peaks, each representing a different population of water. These three water populations are categorized based on the peak areas derived from T_2_ relaxation: bound water P_2b_ (relaxation time constant T_2b_, 1–10 ms), that firmly binds to muscle proteins; immobilized water P_21_ (T_21_, 10–100 ms), located within the interior spaces of muscle fibers; and free water P_22_ (T_22_, 100–1000 ms) in the exterior space of muscle fibers [62]. The water-holding capacity of muscles is thought to be intimately linked to variations in T_2_ relaxation duration, which directly reflect changes in muscle fiber structure and water distribution [63]. The water distribution in fresh meat and the three treatment groups on days 6 and 12 is depicted in Figure 5. The relaxation time of T_2b_ bound to water increased and shifted to the left, in the −4 °C + SMF treatment group on day six, indicating a reduction in the retention of water by muscle proteins [63]. In contrast, samples treated with −4 °C + SEF + SMF demonstrated superior performance, exhibiting the lowest degree of water migration among all groups. The relaxation time of T_2_ in the −4 °C + SEF + SMF treatment group showed no significant difference compared to fresh beef, although the −4 °C + SMF treatment successfully stabilized the relaxation times of T_21_ and T_22_. However, as storage time increased, oxidative denaturation of myofibrillar proteins reduced the water-binding capacity, leading to a decline in the stability of the T2 relaxation time. The degree of muscle fiber integrity in the three moisture groups of beef significantly decreased on the 12th day, with notable changes in T_2_ relaxation times observed in the −4 °C treatment group. However, only T_21_ changed significantly in the −4 °C + SEF + SMF and −4 °C + SMF treatment groups compared to fresh beef, suggesting that both SMF and SEF + SMF were effective in preserving the beef’s moisture content throughout the 12-day storage period. The magnetic field likely strengthens protein–water interactions, modulates water distribution, and improves the water-holding capacity of myofibrillar protein, thereby suppressing water migration [64]. Xu et al. [65] also found that applying electrostatic fields to stored pork delayed the deterioration of muscle fibers and reduced water migration during frozen pork storage.

Figure 5C displays the peak area ratios of the three water populations. The peak area ratios in the −4 °C + SEF + SMF and −4 °C + SMF treatment groups show significant differences on the third day. This is partially because of the absence of ice crystals [66] and partly on the ability of SEF and SMF to prevent water from migrating to free water [67]; the combined use can enhance the inhibition of immobilized water migration to free water. A pattern of an initial decline followed by an increase in P_22_ was observed across all three groups, with the most pronounced trend occurring in the −4 °C treatment group. This phenomenon may result from the formation of ice crystals during low-temperature storage, which punctures the muscle fiber cells of beef and converts immobilized water within the cells to free water during thawing. In later stages of storage, the bacterial activity and other factors further compromise the structural integrity of the beef muscle fibers, leading to the migration of bound water to immobilized water. The reduced ability of the muscle fibers to retain immobilized water causes further migration to fixed water, thereby increasing P_22_.

In the magnetic resonance imaging (MRI) false-color image, regions with greater water concentrations are represented by more noticeable red colors, while areas with lower water concentrations are indicated by more noticeable blue colors. The effect of various treatment groups on MRI is displayed in Figure 5D. The red distribution in the −4 °C + SEF + SMF treatment group appears to be more consistent compared to the −4 °C + SMF and −4 °C treatment groups. This observation warrants further investigation, as it could be directly connected to the magneto–electric coupling process.

## 4. Conclusions

The SMF effectively lowers the supercooling point of beef, enabling it to remain unfrozen at lower temperatures, while sufficient intensity of SEF promotes water nucleation in beef. This leads to the rapid formation of more and smaller ice crystals, minimizing their impact on the beef quality. Compared to using a static magnetic field alone, magneto–electric coupling maintains beef quality at a fresher level. Magneto–electric coupling has a greater impact on preserving beef quality by maintaining pH value, reducing protein and lipid oxidation, controlling total bacterial count, preserving shear force, inhibiting calpain activity, and maintaining water distribution. This storage method’s design allows beef to be stored at lower temperatures while offering a theoretical foundation for prolonging the shelf life and preserving the quality of cold, fresh beef. Furthermore, adding SEF during long-term low-temperature storage may mitigate quality deterioration in beef caused by supercooling failure. Ultimately, the study found that magneto–electric coupling might result in a more even distribution of water molecules in beef, a phenomenon worth exploring in subsequent research. The results indicate that the magneto–electric coupling can significantly improve the quality of beef under refrigeration conditions and has broad application prospects in meat preservation.

As different cuts with varying fat content and muscle composition may respond differently to the supercooling process, we are digging deeper into the mechanism by which fat content influences the supercooling point, investigating the effects of magneto–electric coupled treatment on beef with varying fat contents. This will provide a precise theoretical basis and technical targets for developing differentiated preservation strategies.

## Data Availability

The authors declare that the data supporting the findings of this study are available within the paper. Should any raw data files be needed in another format they are available from the corresponding author upon reasonable request.

## Abbreviations

The following abbreviations are used in this manuscript:

SMF	Static magnetic field
SEF	Static electric field
TVC	Total viable counts
BLL	Beef longissimus lumborum
PE	Polyethylene film
TVB-N	Total volatile basic nitrogen
TBARS	Thiobarbituric acid active substance
TCA	Trichloroacetic acid
EDTA	Ethylene diamine tetra-acetic acid
HVEF	High voltage electrostatic field
CHVEF	Continuous high-voltage electrostatic fields
IHVEF	Intermittent high voltage electrostatic field
SHVEF	Static high voltage electrostatic field
MDA	Malondialdehyde
LF-NMR	Low-field nuclear magnetic resonance
MRI	Magnetic resonance imaging
PEF	Pulsed electric fields
